# Supplementary material for: Infants make moral character inferences in multi-agent social interactions
Source: Commun Psychol. 2026 Feb 12;4:51. doi: 10.1038/s44271-026-00417-8 (PMC13008774; doi:10.1038/s44271-026-00417-8)
Supplement: Supplementary file 2 — Supplementary Information [file 44271_2026_417_MOESM2_ESM.pdf]

Supplementary Information

Supplementary Methods 1: Sensitivity Analyses for Experiment 1

As we did not perform an a priori power analysis in Experiment 1, we chose to conduct a sensitivity analysis to determine if our studies were sufficiently powered to detect the effect sizes that we found. For each of our significant effects, we used G*Power 3.1.9.7 to calculate the minimum effect size necessary to achieve 80% power for each of our analyses and for our sample size. We also computed the power of each of our significant analyses by treating the obtained effect size as the population effect size. These analyses are reported in Supplemental Table 1.

Supplemental Table 1: Sensitivity Analysis for Experiment 1

Analysis	Computed Effect Size	Computed Power	Effect Size Required for 80% power	Interpretation
Main ANOVA interaction	$\eta^2_p = .12$	1.00	$\eta^2_p = .019$	Sufficiently Powered
Aggressor t-test	$d = .43$	.65	$d = .51$	Under Powered
Protector t-test	$d = .63$	.93	$d = .51$	Sufficiently Powered
Victim t-test	$d = .37$	.52	$d = .51$	Under Powered
Aggressor - Protector	$d = 1.10$	.99	$d = .71$	Sufficiently Powered
Aggressor - Victim	$d = .88$	.93	$d = .71$	Sufficiently Powered
Aggressor - Random	$d = .86$	.92	$d = .71$	Sufficiently Powered
Protector - Bystander	$d = .82$	.90	$d = .71$	Sufficiently Powered
Victim - Bystander	$d = .65$	.73	$d = .71$	Under Powered
Bystander - Random	$d = .62$	.68	$d = .71$	Under Powered

Examining the effect sizes necessary to achieve 80% power, our main analysis of interest, the mixed ANOVA testing the interaction between condition and test trial type was sufficiently powered to detect a small effect size, the paired sample t-tests conducted in each condition were sufficiently powered to detect medium effect sizes, and the between condition comparisons using two sample t-tests were sufficiently powered to detect medium to large effect sizes (80% power and size of effects were determined based on guidelines set by Cohen, 1992 and Cohen, 1962).

If we assume that the population effect sizes are equal to the sample effect sizes that we obtain, then most of our analyses are sufficiently powered. In particular, the analysis most pertinent to our hypotheses, the condition by test trial type ANOVA, was very well powered. There were, however, some statistical tests that were underpowered.

In conducting this sensitivity analysis, we acknowledge the limitations of power analyses conducted after data has been collected (Levine & Ensom, 2001; Zhang et al., 2019; O'Keefe, 2007), and before data has been collected (Albers & Lakens, 2018). However, we believe that the analyses here still provide value considering that many studies published in psychology are underpowered, and because they may assist in providing a more balanced view of the results (Cohen, 1962; O'Keefe, 2007).

Supplementary Methods 2: Familiarization Trials

Experiment 1

To determine whether condition and/or trial number affected infants' attention on familiarization a two-way mixed ANOVA with condition as the between-subjects factor and trial numbers as the within-subjects factor was conducted. This analysis revealed a main effect of familiarization trial number ($F(3,465) = 23.20$, $p < .001$, $\eta^2_p = .13$, 95%CI [.08, .18]) such that

looking time decreased on each subsequent familiarization trial. There was a significant effect of condition ($F(4,155) = 4.09$, $p = .003$, $\eta^2_p = .10$, 95% CI [.01, .18]) and no significant interaction ($F(12,465) = 1.42$, $p = .15$, $\eta^2_p = .04$, 95% CI [.00, .07). These familiarization trials are displayed in Supplementary Figure 1.

Follow-up analyses revealed that the significant effect of condition was mainly driven by differences between the Protector Condition and the other four conditions (Aggressor: $F(1,62) = 7.84$, $p = .006$, $\eta^2_p = .11$, 95% CI [.01, .27]; Victim: $F(1,62) = 4.50$, $p = .038$, $\eta^2_p = .07$, 95% CI [.00, .21]; Bystander: $F(1,62) = 11.79$, $p = .001$, $\eta^2_p = .16$, 95% CI [.03, .33]; Random: $F(1,62) = 12.10$, $p < .001$, $\eta^2_p = .16$, 95% CI [.03, .33]). While the difference in looking towards familiarization trials between the Protector Condition and the Bystander and Random Movement Conditions was not particularly surprising given that infants saw different familiarization trials, the differences between the Protector Condition and the Aggressor and Victim Conditions were more unexpected. To ensure that differences in infants looking in the test trials unaffected by looking in the familiarization trials, we conducted a linear regression to determine if infants total looking time to familiarization trials predicted their proportion score, while controlling for condition. There was no effect of familiarization looking on infants looking proportion in test trials ($t(154) = 1.48$, $p = 0.14$). Similar analyses examining the effect of familiarization on difference scores (fair – unfair looking time), not controlling for condition, and investigating the interaction between condition and familiarization looking all did not find significant effects.

Supplementary Figure 1
Experiment 1 Familiarization Trials

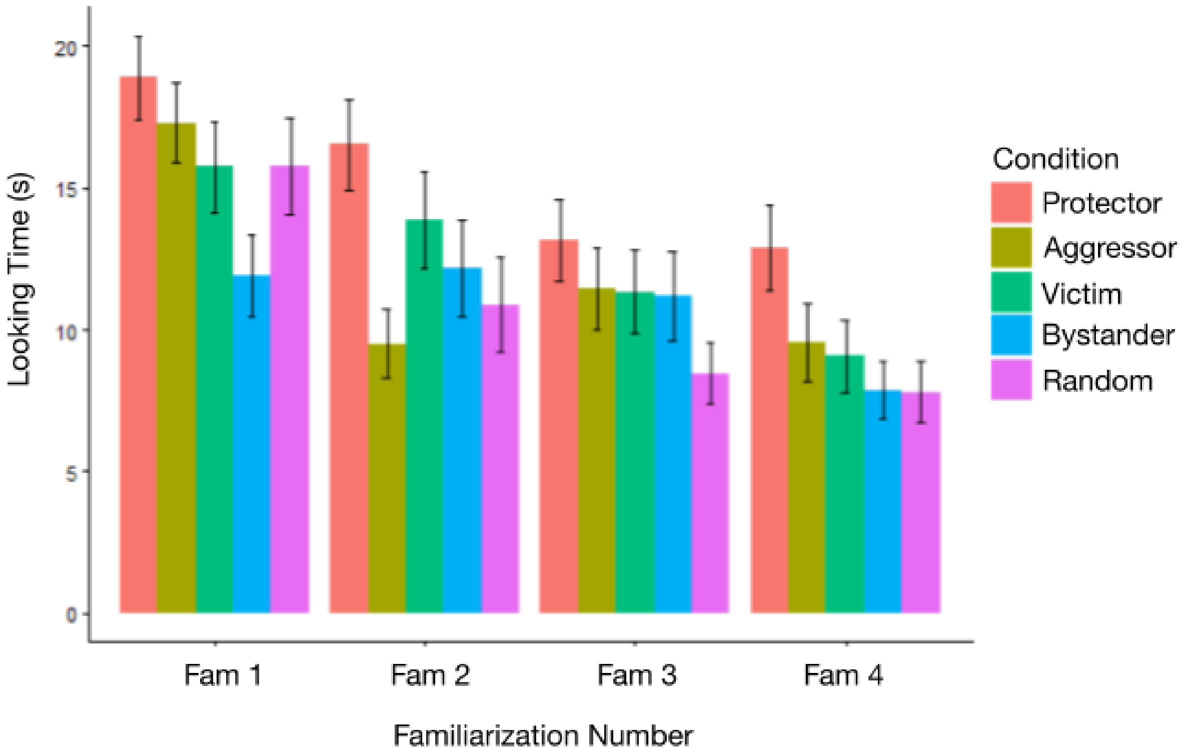

Note. $n = 32$ per condition. Error bars represent standard error.

Experiment 2

To ensure that there were no differences in infants looking to familiarization trials, a two-way mixed ANOVA with condition as the between-subjects factor and trial numbers as the within-subjects factor was conducted. This analysis revealed a main effect of familiarization trial number ($F(3,376) = 8.92$, $p < .001$, $\eta^2_p = .06$, 95%CI [.02, .12]) such that looking time decreased on each subsequent familiarization trial. There was no significant effect of condition ($F(1,376) = .11$, $p = .74$, $\eta^2_p = .00$, 95%CI [.00, .01]) nor was there a significant interaction ($F(3,376) = .56$, $p = .64$, $\eta^2_p = .00$, 95%CI [.00, .02]). These familiarization trials are displayed in Supplementary Figure 2.

Supplementary Figure 2

Experiment 2 Familiarization Trials

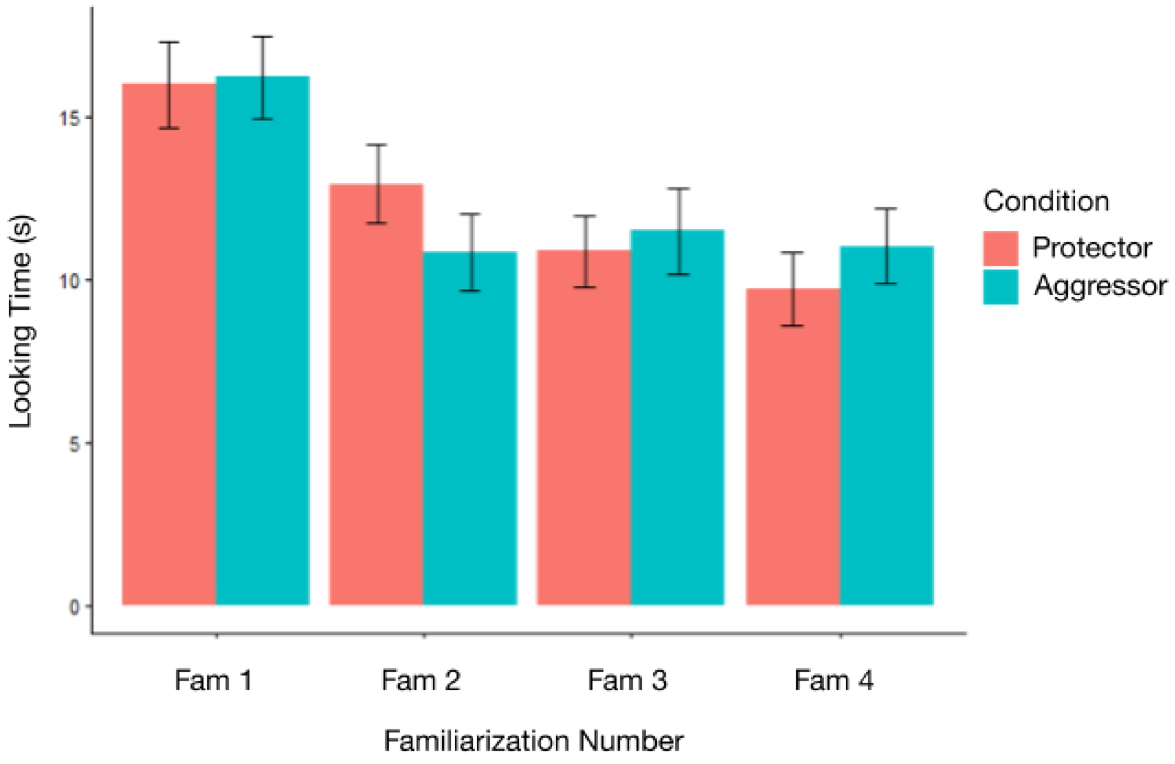

Note. $n = 48$ per condition. Error bars represent standard error.

Supplementary Note 1: Exploratory Comparisons Using Difference Scores

In the main text, comparisons between conditions were conducted using proportion scores. An alternative method to conduct these analyses is using raw difference scores, calculated by subtracting the amount of time infants looked at the unfair test trial from the amount of time infants looked at the fair test trial (difference score = fair – unfair looking time). Using this method, all comparisons that were significant in the main text remain significant except for the comparison between the Bystander and the Random Movement Conditions, which becomes marginally significant. This analysis, and an equivalent analysis using ANOVAs, are reported in Supplementary Table 2 and Supplementary Table 3.

Supplementary Table 2: Experiment 1 Exploratory Analyses Using Difference Scores

Agent	Protector	Victim	Random	Bystander	Aggressor
Protector	-	d = .16<br>95% CI<br>[-.34, .65]	d = .21<br>95% CI<br>[-.28, .71]	**d = .76<br>95% CI<br>[.24, 1.26]**	**d = 1.06<br>95% CI<br>[.53, 1.58]**
Victim	t = .62<br>p = .54<br>$p_{adj.}$ = .97	-	d = .05<br>95% CI<br>[-.44, .54]	**d = .53<br>95% CI<br>[.03, 1.03]**	**d = .80<br>95% CI<br>[.29, 1.31]**
Random	t = .86<br>p = .39<br>$p_{adj.}$ = .91	t = .21<br>p = .83<br>$p_{adj.}$ = .99	-	d = .47<br>95% CI<br>[-.02, .97]	**d = .75<br>95% CI<br>[.24, 1.26]**
Bystander	**t = 3.02<br>p = .004<br>$p_{adj.}$ = .047**	**t = 2.11<br>p = .038<br>$p_{adj.}$ = .20**	t = 1.90<br>p = .06<br>$p_{adj.}$ = .30	-	d = .30<br>95% CI<br>[-.19, .79]
Aggressor	**t = 4.23<br>p < .001<br>$p_{adj.}$ = .001**	**t = 3.21<br>p = .002<br>$p_{adj.}$ = .010**	**t = 3.00<br>p = .004<br>$p_{adj.}$ = .020**	t = 1.20<br>p = .23<br>$p_{adj.}$ = .77	-

Supplementary Table 3: Experiment 1 Exploratory Analyses Using ANOVAs

Agent	Protector	Victim	Random	Bystander	Aggressor
Protector	-	η^2_p = .00<br>95% CI<br>[.00, .10]	η^2_p = .01<br>95% CI<br>[.00, .11]	**η^2_p = .13<br>95% CI<br>[.02, .29]**	**η^2_p = .22<br>95% CI<br>[.07, .39]**
Victim	F = .39<br>p = .54	-	η^2_p = .00<br>95% CI<br>[.00, ,06]	**η^2_p = .07<br>95% CI<br>[.00, .21]**	**η^2_p = .14<br>95% CI<br>[.02, .31]**
Random	F = .74<br>p = .39	F = .05<br>p = .83	-	η^2_p = .05<br>95% CI<br>[.00, ,20]	**η^2_p = .13<br>95% CI<br>[.01, .29]**

Bystander	***F* = 9.12**<br>***p* = .004**	***F* = 4.47**<br>***p* = .038**	*F* = 3.60<br>*p* = .06	-	η^2_p = .02<br>95% CI<br>[.00, .14]
Aggressor	***F* = 17.85**<br>***p* < .001**	***F* = 10.32**<br>***p* = .002**	***F* = 9.03**<br>***p* = .004**	*F* = 1.44<br>*p* = .23	-

Supplementary Note 2: An alternate data analysis plan

Below, we have included an alternative analysis plan that may further elucidate between condition differences, as suggested by a reviewer. Here, we compare the aggressor and protector conditions directly, followed by the bystander and victim conditions.

Comparing Aggressor and Protector Conditions

To determine whether infants' expectations differed across the Aggressor and Protector conditions we conducted a mixed ANOVA with condition as a between-subjects factor, trial type as a within-subjects factor, and looking time as the dependent variable. This analysis demonstrated that there was a significant test trial type by condition interaction ($F(1, 62) = 9.03$, $p = .004$), and no significant main effects of test trial type or condition. Thus, as predicted, a significant interaction between conditions was obtained.

Comparing Bystander and Victim Conditions

We sought to determine whether the Bystander and Victim Conditions differed from one another. A mixed ANOVA demonstrated that there was a significant test trial type by condition interaction ($F(1,62) = 4.47$, $p = .029$), and no significant main effects of trial type or condition. Thus, as predicted, a significant interaction was obtained.

References

Albers, C., & Lakens, D. (2018). When power analyses based on pilot data are biased: Inaccurate effect size estimators and follow-up bias. *Journal of Experimental Social Psychology*, 74, 187-195. https://doi.org/10.1016/J.JESP.2017.09.004

Cohen, J. (1962). The statistical power of abnormal-social psychological research: A review. *The Journal of Abnormal and Social Psychology*, *65*(3), 145–153. https://doi.org/10.1037/h0045186

Cohen, J. (1992). A Power Primer. *Psychological Bulletin, 112*(1), 155–159.

Levine, M., & Ensom, M. H. H. (2001). Post Hoc Power Analysis: An Idea Whose Time Has Passed? *Pharmacotherapy: The Journal of Human Pharmacology and Drug Therapy*, *21*(4), 405–409. https://doi.org/10.1592/phco.21.5.405.34503

O'Keefe, D. J. (2007). Brief Report: Post Hoc Power, Observed Power, A Priori Power, Retrospective Power, Prospective Power, Achieved Power: Sorting Out Appropriate Uses of Statistical Power Analyses. *Communication Methods and Measures*, *1*(4), 291–299. https://doi.org/10.1080/19312450701641375

Zhang, Y., Hedo, R., Rivera, A., Rull, R., Richardson, S., & Tu, X. M. (2019). Post hoc power analysis: Is it an informative and meaningful analysis? *General Psychiatry*, *32*(4), e100069. https://doi.org/10.1136/gpsych-2019-100069
